# Crystal structure of 3,5-dimeth­oxy-2-[5-(naphthalen-1-yl)-4,5-di­hydro-1*H*-pyrazol-3-yl]phenol

**DOI:** 10.1107/S2056989015016369

**Published:** 2015-09-12

**Authors:** Dongsoo Koh

**Affiliations:** a Department of Applied Chemistry, Dongduk Women’s University, Seoul 136-714, Republic of Korea

**Keywords:** crystal structure, pyrazoline, naphthalene, N—H⋯π and C—H⋯π inter­action, hydrogen bonding

## Abstract

In the title compound, C_21_H_20_N_2_O_3_, the planes of the benzene ring and the naphthalene ring system are inclined to one another by 70.95°, and by 4.99 (6) and 75.93 (5)°, respectively, to the mean plane of the pyrazoline ring. The latter has an envelope conformation with the methine (CH) C atom as the flap. There is an intra­molecular O—H⋯N hydrogen bond that forms an *S*(6) ring motif. In the crystal, mol­ecules are linked by C—H⋯O hydrogen bonds, forming chains along [100]. The chains are linked *via* C—H⋯N hydrogen bonds, forming sheets parallel to the *ab* plane. The sheets are linked by a series of N—H⋯π and C—H⋯π inter­actions forming a three-dimensional structure.

## Related literature   

For the synthesis and biological properties of pyrazoline derivatives, see: Bano *et al.* (2015 ▸); Viveka *et al.* (2015 ▸); Neudorfer *et al.* (2014 ▸); Hwang *et al.* (2013 ▸); Yong *et al.* (2013 ▸); Congiu *et al.* (2010 ▸). For N—H⋯π inter­actions in the crystal structure of 3-(thio­phen-2-yl)-5-*p*-tolyl-4,5-di­hydro-1*H*-pyrazole-1-carbo­thio­amide, see: Naveen *et al.* (2015 ▸). For related structures, see: Zhu *et al.* (2013 ▸); Patel *et al.* (2013 ▸).
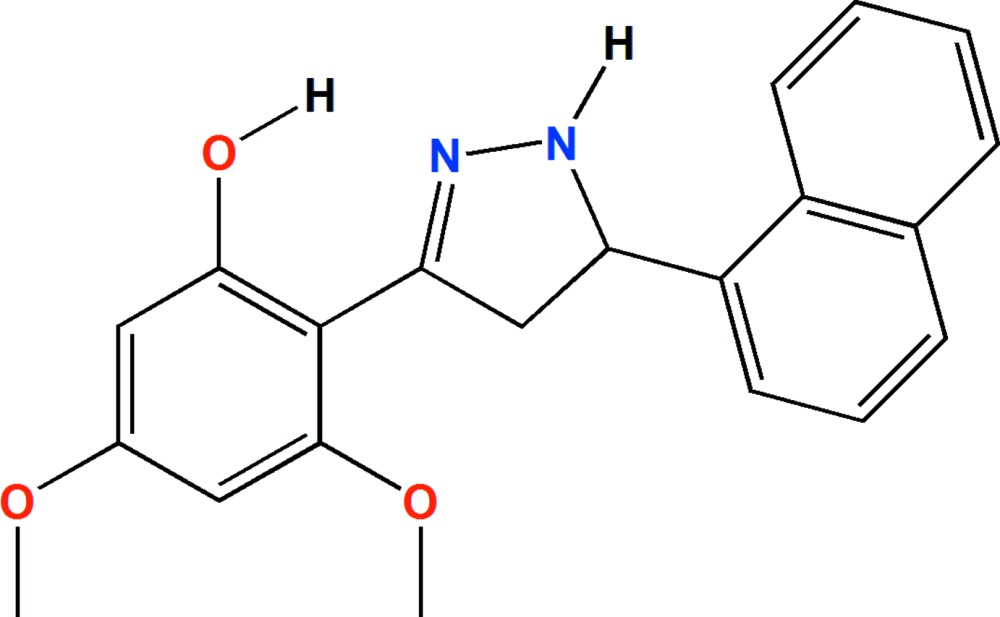


## Experimental   

### Crystal data  

C_21_H_20_N_2_O_3_
*M* *_r_* = 348.39
Triclinic, 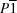

*a* = 7.6248 (5) Å
*b* = 8.6044 (6) Å
*c* = 13.1757 (9) Å
α = 92.832 (4)°
β = 90.777 (3)°
γ = 99.099 (3)°
*V* = 852.30 (10) Å^3^
*Z* = 2
Cu *K*α radiation
μ = 0.74 mm^−1^
*T* = 147 K
0.18 × 0.11 × 0.09 mm

### Data collection  

Bruker Kappa APEX DUO CCD diffractometer
Absorption correction: multi-scan (*SADABS*; Bruker, 2012 ▸) *T*_min_ = 0.698, *T*_max_ = 0.753
21626 measured reflections
2906 independent reflections
2736 reflections with *I* > 2σ(*I*)
*R*_int_ = 0.029

### Refinement  

*R*[*F* ^2^ > 2σ(*F* ^2^)] = 0.036
*wR*(*F* ^2^) = 0.096
*S* = 1.04
2906 reflections
245 parameters
H atoms treated by a mixture of independent and constrained refinement
Δρ_max_ = 0.16 e Å^−3^
Δρ_min_ = −0.22 e Å^−3^

### 

Data collection: *APEX2* (Bruker, 2012 ▸); cell refinement: *SAINT* (Bruker, 2012 ▸); data reduction: *SAINT*; program(s) used to solve structure: *SHELXS97* (Sheldrick, 2008 ▸); program(s) used to refine structure: *SHELXL97* (Sheldrick, 2008 ▸); molecular graphics: *PLATON* (Spek, 2009 ▸) and *Mercury* (Macrae *et al.*, 2008 ▸); software used to prepare material for publication: *SHELXTL* (Sheldrick, 2008 ▸) and *PLATON*.

## Supplementary Material

Crystal structure: contains datablock(s) I, Global. DOI: 10.1107/S2056989015016369/su5198sup1.cif

Structure factors: contains datablock(s) I. DOI: 10.1107/S2056989015016369/su5198Isup2.hkl

Supporting information file. DOI: 10.1107/S2056989015016369/su5198Isup3.cml

**Fig. 1.**
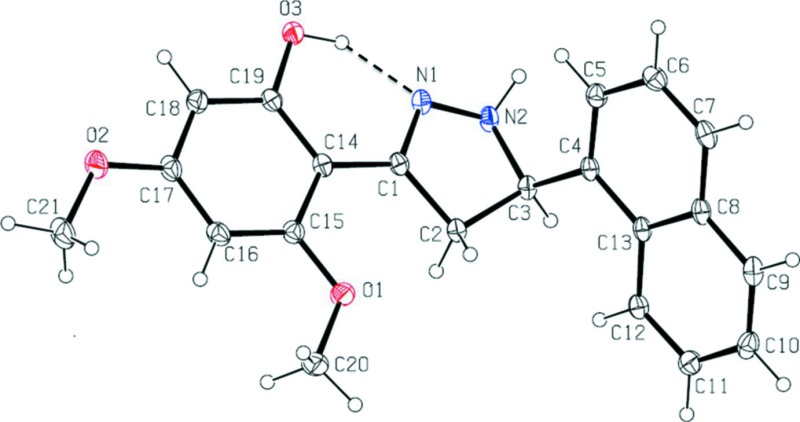
The molecular structure of the title compound, with atom labelling. Displacement ellipsoids are drawn at the 30% probability level.

**Fig. 2.**
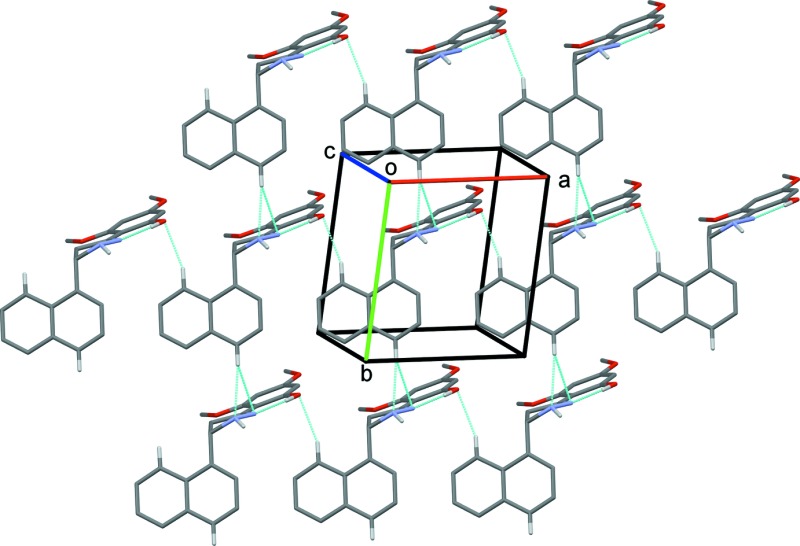
A view along the c axis of the crystal packing of the title compound. The hydrogen bonds are shown as dashed lines (see Table 1).

. DOI: 10.1107/S2056989015016369/su5198fig1.tif
The mol­ecular structure of the title compound, with atom labelling. Displacement ellipsoids are drawn at the 30% probability level.

c . DOI: 10.1107/S2056989015016369/su5198fig2.tif
A view along the *c* axis of the crystal packing of the title compound. The hydrogen bonds are shown as dashed lines (see Table 1).

**Fig. 3.**
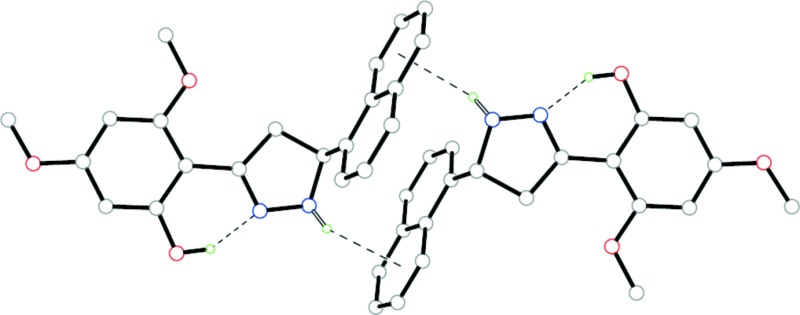
A view of the inversion dimers formed by a pair of N-H···π interactions (dashed lines; see Table 1), in the crystal structure of the title compound.

. DOI: 10.1107/S2056989015016369/su5198fig3.tif
A view of the inversion dimers formed by a pair of N-H⋯π inter­actions (dashed lines; see Table 1), in the crystal structure of the title compound.

**Fig. 4.**
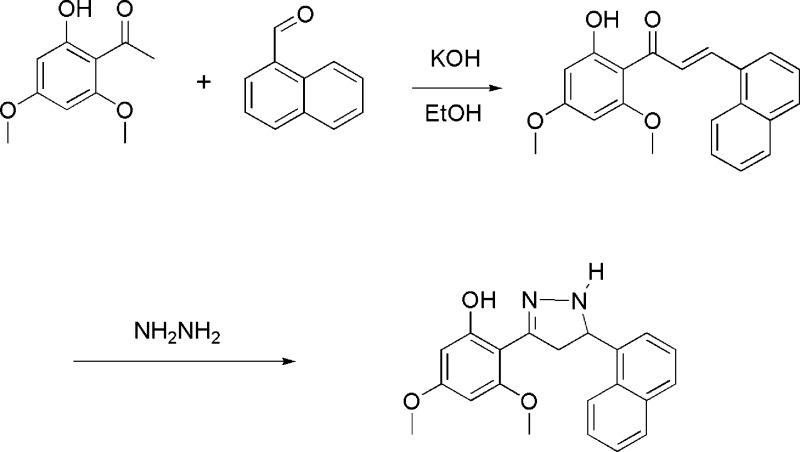
Synthetic scheme for the preparation of the title pyrazoline compound.

. DOI: 10.1107/S2056989015016369/su5198fig4.tif
Synthetic scheme for the preparation of the title pyrazoline compound.

CCDC reference: 1421849

Additional supporting information:  crystallographic information; 3D view; checkCIF report

## Figures and Tables

**Table 1:** Hydrogen-bond geometry (, ) *Cg*2, *Cg*3 and *Cg*4 are the centroids of rings C4C8/C13, C8C13 and C14C19, respectively.

*D*H*A*	*D*H	H*A*	*D* *A*	*D*H*A*
O3H3*O*N1	0.926(18)	1.718(18)	2.5578(12)	149.3(16)
C7H7*A*N2^i^	0.95	2.56	3.4976(16)	171
C12H12*A*O3^ii^	0.95	2.46	3.3663(15)	161
N2H2*N* *Cg*3^iii^	0.898(17)	2.609(17)	3.1906(11)	123.2(12)
C3H3*A* *Cg*2^iii^	1.00	2.84	3.5842(12)	131
C20H20*C* *Cg*4^iv^	0.98	2.93	3.7892(16)	146
C21H21*C* *Cg*4^v^	0.98	2.85	3.6296(17)	137

Symmetry codes: (i) 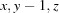; (ii) 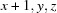; (iii) 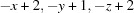; (iv) 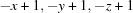; (v) 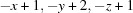.

## supplementary crystallographic information

### S1. Comment

Recent medicinal chemistry researches have been focused on the pyrazoline pharmarcophore. Pyrazolines show a broad spectrum of biological activities including anti­microbial (Bano *et al.*, 2015), anti-inflammatory (Viveka *et al.*, 2015), Alzheimer drugs (Neudorfer *et al.*, 2014) and anti­tumor properties (Congiu *et al.*, 2010). The title pyrazoline compound was synthesized, in continuation of our research program (Hwang *et al.*, 2013), and we report herein on its crystal structure.

The molecular structure of the title compound is shown in Fig. 1. The central pyrazoline ring has an envelope conformation with atom C3 as the flap. The naphthalene and the benzene ring are inclined to the mean plane of the pyrazoline ring by 75.93 (5) and 4.99 (6) °, respectively, and by 70.95 (5) ° to one another. The meth­oxy group at the ortho position of the benzene is almost coplanar with the ring [C20—O1—C15—C16 = 0.8 (2) °], whereas the meth­oxy group at the para position of benzene is slightly twisted from the ring plane [C21—O2—C17—16 = -5.7 (2) °]. The hydroxyl group at the ortho position of the benzene ring makes an intra­molecular O—H···N hydrogen bond to form an S(6)ring motif (Fig. 1 and Table 1).

In the crystal, molecules are linked by C—H···O hydrogen bonds forming chains along [100]. The chains are linked via C—H···N hydrogen bonds forming sheets parallel to the ab plane (Table 1 and Fig. 2). The sheets are linked by a series of N—H···π (Fig. 3) and C—H···π inter­actions (Table 1) forming a three-dimensional structure.

### S2. Synthesis and crystallization

The starting material chalcone was prepared by the previously reported method (Yong *et al.* 2013)and the pyrazoline was obtained by cyclization reaction of the chalcone with NH_2_NH_2_, as illustrated in Fig. 4. To a solution of 6-meth­oxy-2-hy­droxy­aceto­phenone (10 mmol, 1.66g) in 50 ml of ethanol was added 2,3-di­meth­oxy-1-naphthaldehyde (10 mmol, 1.56g) and the temperature was adjusted to around 276-277K in an ice-bath. To the reaction mixture was added 8 ml of 50% (w/v) aqueous KOH solution and the reaction mixture was stirred at room temperature for 20 h. At the end of the reaction, ice water was added to the mixture and it was then acidified with 6N HCl (pH = 3-4). The resulting precipitate was filtered and washed with water and ethanol. The crude solid was purified by recrystallization from ethanol to give the pure chalcone starting material. Excess hydrazine monohydrate (1 ml of 64-65% solution, 13 mmol) was added to a solution of the chalcone compound (5 mmol, 1.52g) in 30 ml anhydrous ethanol, and the solution was refluxed at 360 K for 5 h. The reaction mixture was cooled to room temperature to yield a solid that was then filtered. The crude solids were purified by recrystallization from ethanol to afford the pure pyrazoline title compound as yellow needle-like crystals (yield: 93%; m.p.: 403-403K).

### S3. Refinement

Crystal data, data collection and structure refinement details are summarized in Table 2. The NH and OH H atoms were located in a difference Fourier map and freely refined. The C-bound H atoms were placed in calculated positions and included in the refinement in a riding-model approximation: C–H = 0.95–1.00 Å with U_iso_(H) = 1.5U_eq_(C) for methyl H atoms and 1.2U_eq_(C) for other H atoms.

### Crystal data

**Table:**

C_21_H_20_N_2_O_3_	*Z* = 2
*M**_r_* = 348.39	*F*(000) = 368
Triclinic, *P*1	*D*_x_ = 1.358 Mg m^−^^3^
Hall symbol: -P 1	Cu *K*α radiation, λ = 1.54178 Å
*a* = 7.6248 (5) Å	Cell parameters from 48 reflections
*b* = 8.6044 (6) Å	θ = 6.7–26.4°
*c* = 13.1757 (9) Å	µ = 0.74 mm^−^^1^
α = 92.832 (4)°	*T* = 147 K
β = 90.777 (3)°	Needle, yellow
γ = 99.099 (3)°	0.18 × 0.11 × 0.09 mm
*V* = 852.30 (10) Å^3^	

### Data collection

**Table:**

Bruker Kappa APEX DUO CCD diffractometer	2906 independent reflections
Radiation source: Bruker ImuS	2736 reflections with *I* > 2σ(*I*)
Multi-layer optics monochromator	*R*_int_ = 0.029
φ and ω scans	θ_max_ = 66.4°, θ_min_ = 3.4°
Absorption correction: multi-scan (*SADABS*; Bruker, 2012)	*h* = −9→8
*T*_min_ = 0.698, *T*_max_ = 0.753	*k* = −10→10
21626 measured reflections	*l* = −15→15

### Refinement

**Table:**

Refinement on *F*^2^	Primary atom site location: structure-invariant direct methods
Least-squares matrix: full	Secondary atom site location: difference Fourier map
*R*[*F*^2^ > 2σ(*F*^2^)] = 0.036	Hydrogen site location: inferred from neighbouring sites
*wR*(*F*^2^) = 0.096	H atoms treated by a mixture of independent and constrained refinement
*S* = 1.04	*w* = 1/[σ^2^(*F*_o_^2^) + (0.0556*P*)^2^ + 0.2046*P*] where *P* = (*F*_o_^2^ + 2*F*_c_^2^)/3
2906 reflections	(Δ/σ)_max_ = 0.002
245 parameters	Δρ_max_ = 0.16 e Å^−^^3^
0 restraints	Δρ_min_ = −0.22 e Å^−^^3^

### Special details

**Table:**

Geometry. All e.s.d.'s (except the e.s.d. in the dihedral angle between two l.s. planes) are estimated using the full covariance matrix. The cell e.s.d.'s are taken into account individually in the estimation of e.s.d.'s in distances, angles and torsion angles; correlations between e.s.d.'s in cell parameters are only used when they are defined by crystal symmetry. An approximate (isotropic) treatment of cell e.s.d.'s is used for estimating e.s.d.'s involving l.s. planes.
Refinement. Refinement of *F*^2^ against ALL reflections. The weighted *R*-factor *wR* and goodness of fit *S* are based on *F*^2^, conventional *R*-factors *R* are based on *F*, with *F* set to zero for negative *F*^2^. The threshold expression of *F*^2^ > σ(*F*^2^) is used only for calculating *R*-factors(gt) *etc*. and is not relevant to the choice of reflections for refinement. *R*-factors based on *F*^2^ are statistically about twice as large as those based on *F*, and *R*- factors based on ALL data will be even larger.

### Fractional atomic coordinates and isotropic or equivalent isotropic displacement parameters (Å^2^)

**Table:**

	*x*	*y*	*z*	*U*_iso_*/*U*_eq_	
O1	0.76267 (11)	0.65287 (11)	0.56445 (6)	0.0345 (2)	
O2	0.23406 (12)	0.84718 (12)	0.45432 (7)	0.0395 (2)	
O3	0.33246 (11)	0.79173 (10)	0.79830 (6)	0.0274 (2)	
N1	0.61828 (13)	0.70426 (11)	0.86156 (7)	0.0238 (2)	
N2	0.75961 (13)	0.69468 (12)	0.92877 (7)	0.0257 (2)	
C1	0.66848 (15)	0.68552 (12)	0.76873 (8)	0.0219 (2)	
C2	0.85512 (15)	0.64594 (13)	0.76522 (8)	0.0239 (3)	
H2A	0.9417	0.7366	0.7448	0.029*	
H2B	0.8615	0.5538	0.7182	0.029*	
C3	0.88660 (15)	0.60817 (13)	0.87658 (8)	0.0233 (3)	
H3A	1.0104	0.6549	0.8989	0.028*	
C4	0.85672 (15)	0.43149 (13)	0.89158 (8)	0.0223 (3)	
C5	0.70235 (15)	0.35538 (14)	0.93005 (9)	0.0260 (3)	
H5A	0.6109	0.4141	0.9480	0.031*	
C6	0.67599 (16)	0.19168 (14)	0.94364 (9)	0.0294 (3)	
H6A	0.5673	0.1419	0.9701	0.035*	
C7	0.80537 (17)	0.10457 (14)	0.91907 (9)	0.0281 (3)	
H7A	0.7871	−0.0052	0.9296	0.034*	
C8	0.96708 (16)	0.17698 (13)	0.87785 (8)	0.0244 (3)	
C9	1.10302 (17)	0.08870 (14)	0.85020 (9)	0.0290 (3)	
H9A	1.0856	−0.0214	0.8596	0.035*	
C10	1.25809 (17)	0.15898 (15)	0.81043 (9)	0.0313 (3)	
H10A	1.3475	0.0980	0.7924	0.038*	
C11	1.28539 (16)	0.32194 (15)	0.79617 (9)	0.0295 (3)	
H11A	1.3938	0.3706	0.7687	0.035*	
C12	1.15695 (15)	0.41116 (14)	0.82154 (8)	0.0252 (3)	
H12A	1.1773	0.5209	0.8110	0.030*	
C13	0.99403 (15)	0.34213 (13)	0.86326 (8)	0.0222 (3)	
C14	0.55542 (15)	0.71703 (13)	0.68409 (8)	0.0229 (3)	
C15	0.60542 (15)	0.70520 (14)	0.58125 (9)	0.0257 (3)	
C16	0.50164 (16)	0.74640 (15)	0.50250 (9)	0.0290 (3)	
H16A	0.5377	0.7370	0.4340	0.035*	
C17	0.34405 (16)	0.80168 (15)	0.52538 (9)	0.0291 (3)	
C18	0.28868 (15)	0.81417 (14)	0.62462 (9)	0.0284 (3)	
H18A	0.1801	0.8508	0.6391	0.034*	
C19	0.39277 (15)	0.77291 (13)	0.70265 (9)	0.0240 (3)	
C20	0.82165 (18)	0.64268 (18)	0.46210 (9)	0.0379 (3)	
H20A	0.9381	0.6080	0.4614	0.057*	
H20B	0.8312	0.7464	0.4332	0.057*	
H20C	0.7361	0.5666	0.4216	0.057*	
C21	0.29040 (19)	0.85176 (19)	0.35185 (10)	0.0419 (3)	
H21A	0.2038	0.8948	0.3103	0.063*	
H21B	0.2996	0.7448	0.3256	0.063*	
H21C	0.4066	0.9188	0.3490	0.063*	
H3O	0.420 (2)	0.7700 (19)	0.8426 (14)	0.050 (5)*	
H2N	0.719 (2)	0.6571 (18)	0.9879 (13)	0.039 (4)*	

### Atomic displacement parameters (Å^2^)

**Table:**

	*U*^11^	*U*^22^	*U*^33^	*U*^12^	*U*^13^	*U*^23^
O1	0.0324 (5)	0.0539 (6)	0.0208 (4)	0.0170 (4)	0.0019 (3)	0.0033 (4)
O2	0.0316 (5)	0.0590 (6)	0.0291 (5)	0.0082 (4)	−0.0082 (4)	0.0141 (4)
O3	0.0307 (5)	0.0307 (5)	0.0232 (4)	0.0109 (3)	0.0021 (3)	0.0039 (3)
N1	0.0302 (5)	0.0208 (5)	0.0212 (5)	0.0071 (4)	−0.0036 (4)	0.0012 (4)
N2	0.0334 (5)	0.0256 (5)	0.0198 (5)	0.0108 (4)	−0.0046 (4)	0.0010 (4)
C1	0.0275 (6)	0.0162 (5)	0.0217 (6)	0.0026 (4)	−0.0011 (4)	0.0017 (4)
C2	0.0274 (6)	0.0228 (6)	0.0222 (6)	0.0060 (4)	−0.0021 (4)	0.0036 (4)
C3	0.0275 (6)	0.0213 (6)	0.0217 (6)	0.0062 (4)	−0.0040 (4)	0.0010 (4)
C4	0.0278 (6)	0.0227 (6)	0.0165 (5)	0.0051 (4)	−0.0062 (4)	0.0008 (4)
C5	0.0286 (6)	0.0270 (6)	0.0229 (6)	0.0060 (5)	−0.0027 (4)	0.0009 (4)
C6	0.0317 (6)	0.0287 (6)	0.0260 (6)	−0.0014 (5)	−0.0011 (5)	0.0029 (5)
C7	0.0401 (7)	0.0193 (6)	0.0235 (6)	0.0007 (5)	−0.0065 (5)	0.0019 (4)
C8	0.0335 (6)	0.0221 (6)	0.0178 (5)	0.0057 (5)	−0.0082 (4)	−0.0008 (4)
C9	0.0417 (7)	0.0225 (6)	0.0240 (6)	0.0108 (5)	−0.0091 (5)	−0.0028 (5)
C10	0.0370 (7)	0.0342 (7)	0.0255 (6)	0.0166 (5)	−0.0051 (5)	−0.0041 (5)
C11	0.0299 (6)	0.0355 (7)	0.0236 (6)	0.0076 (5)	−0.0019 (5)	0.0001 (5)
C12	0.0302 (6)	0.0247 (6)	0.0209 (5)	0.0051 (5)	−0.0035 (4)	0.0022 (4)
C13	0.0288 (6)	0.0215 (6)	0.0164 (5)	0.0052 (4)	−0.0065 (4)	0.0003 (4)
C14	0.0261 (6)	0.0200 (6)	0.0222 (6)	0.0022 (4)	−0.0020 (4)	0.0030 (4)
C15	0.0255 (6)	0.0271 (6)	0.0244 (6)	0.0035 (5)	−0.0014 (4)	0.0020 (5)
C16	0.0298 (6)	0.0354 (7)	0.0207 (6)	0.0011 (5)	−0.0020 (5)	0.0039 (5)
C17	0.0264 (6)	0.0323 (7)	0.0274 (6)	−0.0008 (5)	−0.0069 (5)	0.0082 (5)
C18	0.0246 (6)	0.0305 (7)	0.0306 (6)	0.0047 (5)	−0.0019 (5)	0.0065 (5)
C19	0.0269 (6)	0.0205 (6)	0.0240 (6)	0.0017 (4)	0.0005 (4)	0.0037 (4)
C20	0.0376 (7)	0.0549 (9)	0.0234 (6)	0.0137 (6)	0.0056 (5)	0.0037 (6)
C21	0.0459 (8)	0.0534 (9)	0.0260 (7)	0.0051 (6)	−0.0112 (6)	0.0086 (6)

### Geometric parameters (Å, º)

**Table:**

O1—C15	1.3629 (15)	C8—C9	1.4198 (17)
O1—C20	1.4304 (14)	C8—C13	1.4263 (16)
O2—C17	1.3609 (15)	C9—C10	1.3640 (19)
O2—C21	1.4229 (16)	C9—H9A	0.9500
O3—C19	1.3584 (14)	C10—C11	1.4070 (18)
O3—H3O	0.926 (18)	C10—H10A	0.9500
N1—C1	1.2965 (15)	C11—C12	1.3717 (17)
N1—N2	1.4005 (13)	C11—H11A	0.9500
N2—C3	1.4697 (15)	C12—C13	1.4197 (17)
N2—H2N	0.898 (17)	C12—H12A	0.9500
C1—C14	1.4622 (16)	C14—C15	1.4158 (16)
C1—C2	1.5155 (16)	C14—C19	1.4192 (17)
C2—C3	1.5423 (15)	C15—C16	1.3896 (17)
C2—H2A	0.9900	C16—C17	1.3919 (18)
C2—H2B	0.9900	C16—H16A	0.9500
C3—C4	1.5243 (15)	C17—C18	1.3838 (18)
C3—H3A	1.0000	C18—C19	1.3842 (17)
C4—C5	1.3698 (17)	C18—H18A	0.9500
C4—C13	1.4364 (16)	C20—H20A	0.9800
C5—C6	1.4116 (17)	C20—H20B	0.9800
C5—H5A	0.9500	C20—H20C	0.9800
C6—C7	1.3632 (18)	C21—H21A	0.9800
C6—H6A	0.9500	C21—H21B	0.9800
C7—C8	1.4185 (18)	C21—H21C	0.9800
C7—H7A	0.9500		
			
C15—O1—C20	118.10 (9)	C9—C10—H10A	120.0
C17—O2—C21	118.23 (10)	C11—C10—H10A	120.0
C19—O3—H3O	107.0 (11)	C12—C11—C10	120.59 (11)
C1—N1—N2	109.61 (9)	C12—C11—H11A	119.7
N1—N2—C3	108.80 (8)	C10—C11—H11A	119.7
N1—N2—H2N	110.6 (10)	C11—C12—C13	121.07 (11)
C3—N2—H2N	116.0 (10)	C11—C12—H12A	119.5
N1—C1—C14	120.11 (10)	C13—C12—H12A	119.5
N1—C1—C2	111.37 (9)	C12—C13—C8	118.16 (10)
C14—C1—C2	128.25 (10)	C12—C13—C4	122.84 (10)
C1—C2—C3	101.50 (9)	C8—C13—C4	119.00 (10)
C1—C2—H2A	111.5	C15—C14—C19	116.38 (10)
C3—C2—H2A	111.5	C15—C14—C1	122.99 (10)
C1—C2—H2B	111.5	C19—C14—C1	120.48 (10)
C3—C2—H2B	111.5	O1—C15—C16	122.12 (11)
H2A—C2—H2B	109.3	O1—C15—C14	115.85 (10)
N2—C3—C4	114.59 (9)	C16—C15—C14	122.03 (11)
N2—C3—C2	100.95 (9)	C15—C16—C17	119.04 (11)
C4—C3—C2	112.32 (9)	C15—C16—H16A	120.5
N2—C3—H3A	109.6	C17—C16—H16A	120.5
C4—C3—H3A	109.6	O2—C17—C18	115.09 (11)
C2—C3—H3A	109.6	O2—C17—C16	123.79 (11)
C5—C4—C13	119.16 (10)	C18—C17—C16	121.12 (11)
C5—C4—C3	122.00 (10)	C17—C18—C19	119.50 (11)
C13—C4—C3	118.84 (10)	C17—C18—H18A	120.3
C4—C5—C6	121.56 (11)	C19—C18—H18A	120.3
C4—C5—H5A	119.2	O3—C19—C18	116.33 (10)
C6—C5—H5A	119.2	O3—C19—C14	121.73 (10)
C7—C6—C5	120.49 (11)	C18—C19—C14	121.93 (11)
C7—C6—H6A	119.8	O1—C20—H20A	109.5
C5—C6—H6A	119.8	O1—C20—H20B	109.5
C6—C7—C8	120.33 (11)	H20A—C20—H20B	109.5
C6—C7—H7A	119.8	O1—C20—H20C	109.5
C8—C7—H7A	119.8	H20A—C20—H20C	109.5
C7—C8—C9	121.52 (11)	H20B—C20—H20C	109.5
C7—C8—C13	119.45 (11)	O2—C21—H21A	109.5
C9—C8—C13	119.03 (11)	O2—C21—H21B	109.5
C10—C9—C8	121.24 (11)	H21A—C21—H21B	109.5
C10—C9—H9A	119.4	O2—C21—H21C	109.5
C8—C9—H9A	119.4	H21A—C21—H21C	109.5
C9—C10—C11	119.91 (11)	H21B—C21—H21C	109.5
			
C1—N1—N2—C3	−21.89 (12)	C9—C8—C13—C4	−179.78 (9)
N2—N1—C1—C14	−169.55 (9)	C5—C4—C13—C12	179.10 (10)
N2—N1—C1—C2	4.94 (12)	C3—C4—C13—C12	−0.14 (15)
N1—C1—C2—C3	12.50 (12)	C5—C4—C13—C8	−1.20 (15)
C14—C1—C2—C3	−173.56 (10)	C3—C4—C13—C8	179.56 (9)
N1—N2—C3—C4	−92.86 (11)	N1—C1—C14—C15	177.33 (10)
N1—N2—C3—C2	28.06 (11)	C2—C1—C14—C15	3.86 (18)
C1—C2—C3—N2	−23.31 (10)	N1—C1—C14—C19	1.95 (16)
C1—C2—C3—C4	99.21 (10)	C2—C1—C14—C19	−171.52 (10)
N2—C3—C4—C5	14.33 (15)	C20—O1—C15—C16	0.79 (17)
C2—C3—C4—C5	−100.10 (12)	C20—O1—C15—C14	−178.39 (11)
N2—C3—C4—C13	−166.45 (9)	C19—C14—C15—O1	179.38 (10)
C2—C3—C4—C13	79.12 (12)	C1—C14—C15—O1	3.83 (17)
C13—C4—C5—C6	0.78 (16)	C19—C14—C15—C16	0.20 (17)
C3—C4—C5—C6	180.00 (10)	C1—C14—C15—C16	−175.35 (10)
C4—C5—C6—C7	0.37 (17)	O1—C15—C16—C17	−178.84 (11)
C5—C6—C7—C8	−1.09 (17)	C14—C15—C16—C17	0.29 (18)
C6—C7—C8—C9	−179.07 (10)	C21—O2—C17—C18	174.16 (11)
C6—C7—C8—C13	0.64 (16)	C21—O2—C17—C16	−5.72 (18)
C7—C8—C9—C10	179.88 (10)	C15—C16—C17—O2	179.07 (11)
C13—C8—C9—C10	0.16 (16)	C15—C16—C17—C18	−0.81 (18)
C8—C9—C10—C11	0.00 (17)	O2—C17—C18—C19	−179.07 (10)
C9—C10—C11—C12	−0.28 (17)	C16—C17—C18—C19	0.82 (18)
C10—C11—C12—C13	0.38 (17)	C17—C18—C19—O3	178.46 (10)
C11—C12—C13—C8	−0.20 (16)	C17—C18—C19—C14	−0.30 (18)
C11—C12—C13—C4	179.50 (10)	C15—C14—C19—O3	−178.89 (10)
C7—C8—C13—C12	−179.78 (9)	C1—C14—C19—O3	−3.22 (16)
C9—C8—C13—C12	−0.07 (15)	C15—C14—C19—C18	−0.20 (17)
C7—C8—C13—C4	0.50 (15)	C1—C14—C19—C18	175.47 (10)

### Hydrogen-bond geometry (Å, º)

Cg2, Cg3 and Cg4 are the centroids of rings C4–C8/C13, C8–C13 and C14–C19, respectively.

**Table:**

*D*—H···*A*	*D*—H	H···*A*	*D*···*A*	*D*—H···*A*
O3—H3*O*···N1	0.926 (18)	1.718 (18)	2.5578 (12)	149.3 (16)
C7—H7*A*···N2^i^	0.95	2.56	3.4976 (16)	171
C12—H12*A*···O3^ii^	0.95	2.46	3.3663 (15)	161
N2—H2*N*···*Cg*3^iii^	0.898 (17)	2.609 (17)	3.1906 (11)	123.2 (12)
C3—H3*A*···*Cg*2^iii^	1.00	2.84	3.5842 (12)	131
C20—H20*C*···*Cg*4^iv^	0.98	2.93	3.7892 (16)	146
C21—H21*C*···*Cg*4^v^	0.98	2.85	3.6296 (17)	137

Symmetry codes: (i) *x*, *y*−1, *z*; (ii) *x*+1, *y*, *z*; (iii) −*x*+2, −*y*+1, −*z*+2; (iv) −*x*+1, −*y*+1, −*z*+1; (v) −*x*+1, −*y*+2, −*z*+1.
