# Supplementary figures and images for: Crystal structure of 3,5-dimeth­oxy-2-[5-(naphthalen-1-yl)-4,5-di­hydro-1H-pyrazol-3-yl]phenol
Source: Acta Crystallogr E Crystallogr Commun. 2015 Sep 12;71(Pt 10):o708–9. doi: 10.1107/S2056989015016369 (PMC4647439; doi:10.1107/S2056989015016369)

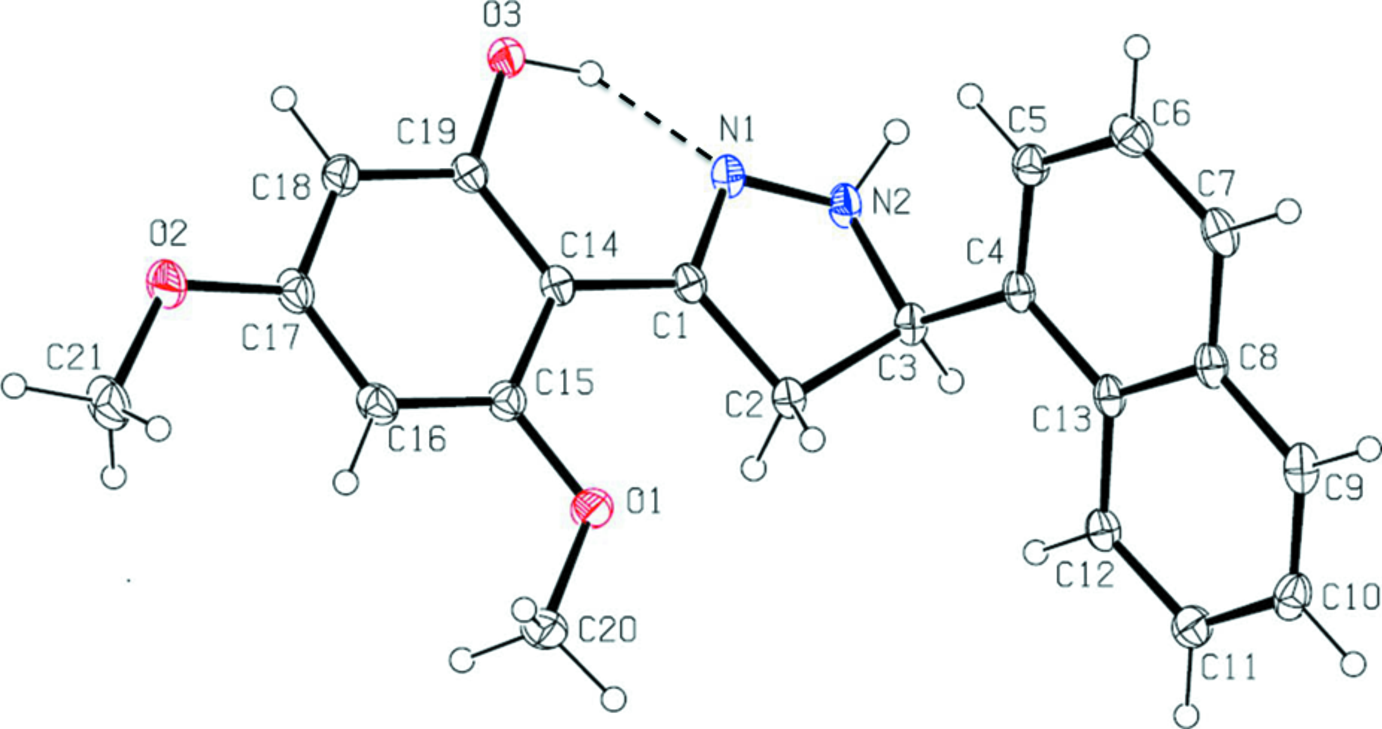

Supplement: Supplementary file 4 [file e-71-0o708-fig1.tif]

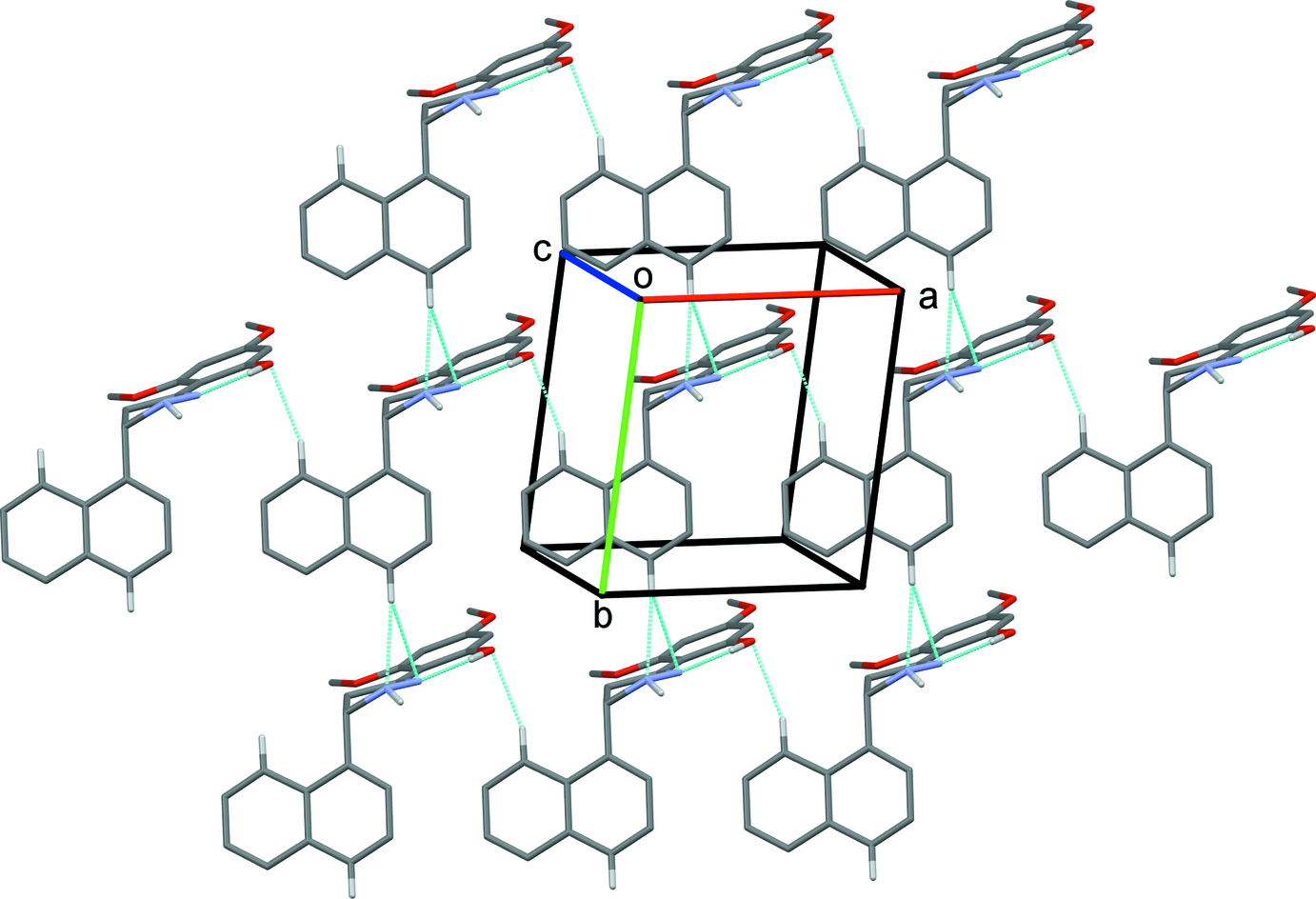

Supplement: Supplementary file 5 [file e-71-0o708-fig2.tif]

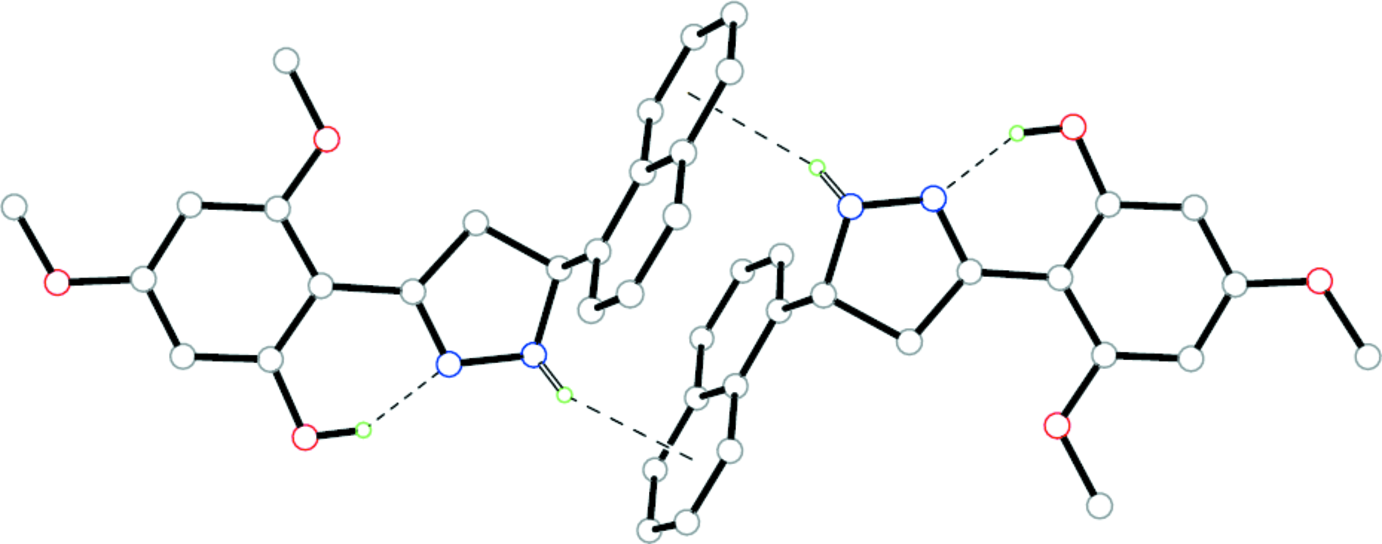

Supplement: Supplementary file 6 [file e-71-0o708-fig3.tif]
